# Prevalence of vancomycin-resistant Enterococcus fecal colonization among kidney transplant patients

**DOI:** 10.1186/1471-2334-6-133

**Published:** 2006-08-22

**Authors:** Maria Cecília S Freitas, Alvaro Pacheco-Silva, Dulce Barbosa, Suzane Silbert, Hélio Sader, Ricardo Sesso, Luis Fernando A Camargo

**Affiliations:** 1 Department of Medicine, Division of Nephrology, Universidade Federal de São Paulo (UNIFESP), Hospital do Rim e Hipertensão, Brazil; 2 Special Clinical Microbiology Laboratory (LEMC), Division of Infectious Diseases-UNIFESP, Brazil; 3 Infectious Diseases Unit, Hospital do Rim e Hipertensão, Universidade Federal de São Paulo, Brazil; 4 Department of Medicine, Division of Nephrology and Infectious Diseases, Universidade Federal de São Paulo, Hospital do Rim e Hipertensão, SP, Brazil

## Abstract

**Background:**

End stage renal disease patients are at risk of Vancomycin-Resistant Enterococcus (VRE) infections. The first reports of VRE isolation were from hemodialysis patients. However, to date, VRE fecal colonization rates as well as associated risk factors in kidney transplant patients have not yet been established in prospective studies.

**Methods:**

We collected one or two stool samples from 280 kidney transplant patients and analysed the prevalence of VRE and its associated risk factors. Patients were evaluated according to the post-transplant period: group 1, less than 30 days after transplantation (102 patients), group 2, one to 6 months after transplantation (73 patients) and group 3, more than 6 months after transplantation (105 patients).

**Results:**

The overall prevalence rate of fecal VRE colonization was 13.6% (38/280), respectively 13.7% for Group 1, 15.1% for group 2 and 12.4% for group 3. *E. faecium *and *E. faecalis *comprised 50% of all VRE isolates. No immunologic variables were clearly correlated with VRE colonization and no infections related to VRE colonization were reported.

**Conclusion:**

Fecal VRE colonization rates in kidney transplant patients were as high as those reported for other high-risk groups, such as critical care and hemodialysis patients. This high rate of VRE colonization observed in kidney transplant recipients may have clinical relevance in infectious complications.

## Background

Despite improvements in surgical techniques and development of new immunosuppressive drugs, infections remain the second most frequent cause of mortality in kidney transplant patients.

*Enterococci *are frequent causative agents of both nosocomial and community acquired infection in transplant patients, including bacteremia, urinary tract infections and surgical site infections [1]. In the past two decades, resistance to glycopeptides has emerged in an epidemic fashion and is now endemic in many countries. One of the first descriptions of Vancomycin-Resistant Enterococcus (VRE) was in patients with end stage renal disease (ESRD) [2] and such patients seem particularly susceptible to colonization.

The prevalences of VRE colonization and infection are especially high among intensive care unit (ICU) patients and in oncology and organ transplant wards. The prevalence of VRE in liver transplant patients range from 3.4 % in patients in the waiting list [3] to as high as 44 % after transplantation [4].

Kidney transplant patients may be prone to developing high rates of VRE colonization and infection due to frequent use of antibiotics, particularly vancomycin both before and after transplantation. However, although kidney transplantation is the solid organ transplantation most frequently performed, little is known about prevalence and risk factors for fecal colonization by VRE among these patients.

The aim of this study was therefore to evaluate the prevalence of fecal colonization by VRE among kidney transplant recipients in three different post-transplantation periods and identify risk factors related to fecal VRE colonization among kidney transplant recipients.

## Methods

This study was conducted at the Hospital do Rim e Hipertensão (HRH), affiliated to the Federal University of São Paulo (UNIFESP), Brazil, and was approved by the Institutional Ethics Committee. All patients gave a written informed consent to participate in the study.

One gram of IV cephalothin is administered immediately before surgery and every 6 hours for the first 48 hours. Trimethoprim-sulfamethoxazole 80 mg-400 mg is prescribed in a single daily oral dose for 6 months, for urinary tract infections prophylaxis.

The immunosuppressive maintenance regimen plan consists mainly of azathioprine, prednisone and cyclosporin or alternatively tacrolimus, rapamycin and mycophenolate Mofetil, in different combinations. For anti-rejection treatment, high doses of corticosteroids, antilymphocyte globulin and monoclonal antibodies are used for short periods. Ganciclovir is not given as routine prophylaxis but is used for the treatment of patients with positive CMV antigenemia.

### Patients

VRE colonization was investigated in three different groups of patients, in three distinct post-transplant periods, as follows: 1) Recent recipients, up to 30 days after transplantation (group 1); 2) Between 1 and 6 months of transplantation (group 2); 3) After 6 months of transplantation (group 3). We controlled patient register numbers in order to avoid the same patient being evaluated in different groups.

In Group 1, patients first samples were collected while they were still in hospital, shortly after transplantation and the second sample for the same patient was collected occasionally after discharge. Groups 2 and 3 patients were evaluated during routine follow up appointment in an outpatient facility.

### VRE colonization evaluation

For each patient we planned to collect two stool specimens with a one-week-interval. For Group 1 patients, the first specimen was collected within one week of transplantation. The specimens were processed at the Special Clinical Microbiology Laboratory (LEMC) of the Infectious Diseases Department of UNIFESP. Patients were considered to be colonized by VRE if species of VRE were identified in at least one of the stool samples tested.

Stool specimens were collected in sterile receptacles and plated on media specifically selective for VRE (azide blood agar, OXOID-England, with 6 μg/mL of vancomycin)[5]. *Enterococcus *isolates were identified to the species level with the conventional biochemical tests as described by Facklam, Sham and Teixeira [6]. All samples were investigated by disk diffusion NCCLS [7] for resistance to ampicillin, vancomycin, teicoplanin, streptomycin and gentamicin. MICs of vancomycin, teicoplanin, ampicillin, streptomycin and gentamicin were determined by Etest (AB Biodisk, Solna, Sweden) for isolates with vancomycin inhibition zones ≤ 16 mm. Isolates were categorized according to the National Committee for Clinical Laboratory Standards (NCCLS) breakpoints [7]. ATTC strains *Staphylococcus aureus *29213 and *E. faecalis *29212 were used as controls.

### Demographic data and risk factors

The following variables were obtained and defined for each patient, by direct interviews and chart review: name; gender; age; race; primary renal disease; modality of renal replacement therapy employed immediately before transplantation (hemodialysis, peritoneal dialysis or conservative treatment); the total length of time in months on dialysis therapy before transplantation, considering all the methods and treatments. For Group 1 patients, antibiotic consumption (including vancomycin use) was not analyzed, since data was not considered accurate using direct interviews.

The following medical reports regarding transplantation were collected: by chart reviews seropositivity to hepatitis B and C at the time of the transplantation, using Ag Hbs and anti-HCV antibodies (immunofluorescence); the number of blood transfusions before transplantation; retransplantation; length of stay, calculated as the sum of in hospital days; dialysis therapy after transplantation, if there was more than one hemodialyses session; human leukocyte antigen (HLA) compatibility, divided into two groups: Identical and haploidentical HLA; and distinct HLA living donor and deceased donor; use of monoclonal (anti-CD3) or polyclonal antibodies (OKT3/Thymoglobulin^®^) for induction therapy as well as for treatment of episodes of corticosteroid-resistant acute rejection; pulse therapy using intravenous methylprednisolone for treatment of acute rejection episodes, with one gram daily for 3 to 5 days; use of mycophenolate mofetil; Surgical re-intervention; use of trimethoprim-sulfamethoxazole; use of vancomycin (for groups 2 and 3 only); use of ganciclovir for at least 7 days, as therapy for cytomegalovirus infection and the total duration of hospitalization (total number of in-hospital days from the first day of transplantation to specimen collection, considering all admissions) (Table 3).

### Statistical analysis

To study the risk factors for VRE colonization, the parameters were initially evaluated by univariate analysis, using the chi-square test or the Fisher exact test for categorical variables and the Student's t test for continuous variables. The tests were two-tailed and the significance level was set at p < 0.05. Multivariate logistic regression analysis was performed to evaluate the variables associated with VRE colonization. Both univariate and multivariate analysis were performed for each group separately. The independent variables tested were those with p value less than 0.30 in previous univariate analysis. The statistical program SPSS, version 10.0 for Windows was used in the analyses.

## Results

Between June 2001 and March 2003, 280 kidney transplant recipients were studied: 102 in group 1 (less than 30 days after transplantation), 73 in group 2 (1 to 6 months after transplantation – median 2 months (2–5 months) and 105 in group 3 (more than 6 months after transplantation – median 20 months (6–206 months). The general characteristics of the studied population are shown in Table 1.

**Table 1:** General characteristics of kidney transplant recipients

Number of Patients	280 (100)
Group 1 (less than 30 days after transplantation)	102 (36.4)
Group 2 (1 to 6 months after transplantation)	73 (26)
Group 3 (> 6 months after transplantation)	105 (37.5)
Gender	
Male	163 (58.2)
Race	
White	187 (66.7)
Non-white	93 (33.2)
Primary renal disease	
Indeterminate	137 (48.9)
Hypertension	61 (21.7)
Glomerulonephritis	44 (15.7)
Diabetes Mellitus	11 (3.9)
Others	27 (9.6)
Pre transplant treatment	
Hemodialysis	262 (93.5)
CAPD	12 (4.2)
Conservative	6 (2.1)
Kidney donor	
HLA I and II	160 (57.1)
HLA III and deceased donor	120 (42.8)
Immunosuppressive drugs **	
Mycophenolate mofetil	59 (33.1)
OKT3/Thymoglobulin^®^	13 (7.3)
Methylprednisolone pulse	58 (32.5)
Surgical reexploration	14 (5)
Post-transplantation dialysis	46 (16.4)
Serology	
Hepatitis C virus	31 (11)
Hepatitis B virus	7 (2.5)
Post-transplantation antimicrobial drugs **	117 (65.7)
Vancomycin **	11 (6.1)
Ganciclovir **	18 (10.1)

** Immunosuppressive drugs, post-transplantation antimicrobial drugs, vancomycin and ganciclovir: just for groups 2 and 3.

CAPD = chronic ambulatory peritoneal dialysis; HLA I = identical human leukocyte antigen;

HLA II = haplo-identical human leukocyte antigen; HLA III = distinct human leukocyte antigen;

Values are expressed as N (%).

Two stool specimens were collected from 41 patients in group 1 (40%), 41 patients (56%) in group 2 and 72 patients (69%) in group 3. The remaining patients had only one specimen collected. Group 1 patients first stool samples were collected while patients were still in the hospital. For group 2 and 3 patients first stool samples were collected as they came for regular follow up appointments. The major reason for not collecting a second sample in all groups was that despite offering money support for public transportation, patients did not return for a second consultation only for protocol purposes. In group 1, 60% of specimens were collected more than 7 days apart; and the second sample was excluded from analysis. Group 2 and 3 samples were collected with a maximum interval of 30 days.

A total of 38 patients had VRE fecal colonization among the 280 patients evaluated, with an overall prevalence rate of 13.6%. Of these 38 patients, fourteen (36.8%) were from group 1, eleven (28.9%) from group 2, and thirteen (34.2%) from group 3. Fecal colonization rates were 13.7% in group 1, 15.1% in group 2 and 12.4% in group 3.

Fifty percent of all VRE isolates were either *E. faecalis or E. faecium *(Table 2). *E. gallinarum *colonization accounted for 28.9% of all colonized patients (11 patients). *E. faecium *was isolated from ten patients (26.3%) and *E. faecalis*, from nine patients (23.6%). For 29 out of 38 positive patients, the first collected specimen tested positive, while 9 (24%) had the first specimen negative and the second positive.

**Table 2:** *Enterococcus *species isolated from kidney transplant recipients

Species	Group 1	Group 2	Group 3	Total
*E. faecalis*	8	1	-	9 (23.6)
*E. faecium*	2	3	5	10 (26.3)
*E. gallinarum*	2	5	4	11 (28.9)
*E. casseliflavus*	2	2	3	7 (18.4)
*E. raffinosus*			1	1 (2.6)
Total number of VRE-positive patients	14 (36.8)	11 (28.9)	13 (34.2)	38 (100)

Values are expressed as N or N (%).

**Table 3:** General characteristics of kidney transplant recipients, for group 1, 2 and 3

	**Group 1**			**Group 2**			**Group 3**		
**Characteristic**	**VRE+**	**VRE -**	**p value**	**VRE+**	**VRE -**	**p value**	**VRE+**	**VRE -**	**p value**
Gender									
Male	10(71.4)	58(65.9)	0.769	6(54.5)	35(56.5)	1	4(30.8)	50(54.3)	0.111
Ages, in years	43 ± 14	39 ± 13	0.579	40 ± 10	41 ± 13	0.857	41 ± 11	42 ± 10	0.789
Race									
White	11(78.6)	61(69.3)		7(63.6)	41(66.1)		12(92.3)	55(59.8)	
Non-white	3(21.4)	27(30.6)	0.494	7(63.6)	21(33.9)	0.791	1(7.7)	37(40.3)	0.122
Primary renal disease									
Indeterminate	8(57.1)	45(51.1)		6(54.5)	32(51.6)		7(53.8)	39(42.4)	
Hypertension	3(21.4)	17(19.3)		3(27.3)	16(25.8)			22(23.9)	
Glomerulonephritis	3(21.4)	15(17)			6(9.7)		3(23.1)	17(18.5)	
Diabetes Mellitus		4(4.5)			3(4.8)		1(7.7)	3(3.3)	
Others		7(8)	0.934	2(18.2)	5(8.1)	0.752	2(15.4)	11(12)	0.195
Pre transplant treatment									
Hemodialysis	13(92.9)	79(89.8)		11(100)	58(93.5)		10(76.9)	91(98.9)	
CAPD		7(8)			2(3.2)		2(15.4)	1(1.1)	
Conservative	1(7.1)	2(2.3)	0.269		2(3.2)	1	1(7.7)		0.006
Kidney donor									
HLA I and II	6(42.9)	55(62.5)		5(45.5)	36(58.1)		8(61.5)	50(54.3)	
HLA III and deceased donor	8(57.1)	33(37.5)	0.164	6(54.5)	26(41.9)	0.518	5(38.5)	42(45.7)	0.626
Surgical reexploration	1(7.1)	1(1.1)	0.257	1(9.1)	5(8.1)	1	2(15.4)	4(4.3)	0.16
Mycophenolate mofetil				3(27.3)	31(50)	0.164	4(30.8)	21(22.8)	0.504
OKT3/Thymoglobulin^®^					7(11.3)	0.585		6(6.5)	1
Methylprednisolone pulse				2(18.2)	20(32.3)	0.486	4(30.8)	32(34.8)	1
Pos-transplantation dialysis	4(28.6)	14(15.9)	0.265		13(21)	0.195	1(7.7)	14(15.2)	0.687
Length of hospitalization in days	7 ± 2	8 ± 5	0.714	18 ± 22	17 ± 15	0.617	31 ± 36	23 ± 20	0.223
Serology									
Hepatitis C virus	1(7.1)	7(8)	1		1(1.6)	1	4(30.8)	18(21.4)	0.462
Hepatitis B virus		4(4.5)	1		1(1.6)	1	1(7.7)	1(1.2)	0.236
Antimicrobial drugs				7(63.6)	31(50)	0.519	10(76.9)	69(75)	1
Vancomycin				1(9.1)	1(1.6)	0.28	3(23.1)	6(6.5)	0.081
Ganciclovir					6(9.7)	0.582	2(15.4)	10(10.8)	0.642

CAPD = chronic ambulatory peritoneal dialysis; HLA I = identical human leukocyte antigen;

HLA II = haplo-identical human leukocyte antigen; HLA III = distinct human leukocyte antigen;

Fisher and Student t tests were utilized for mean age and length of hospitalization;

Values are expressed as mean ± SD or N (%).

In the univariate analysis (Table 3), the only variable associated with fecal VRE colonization was chronic ambulatory peritoneal dialysis (CAPD) for group 3 (15.4% *vs *1.1%, p = 0.006).

Multivariate logistic regression disclosed CAPD, hepatitis B seropositivity and vancomycin use (Table 4) as independent risk factors for fecal VRE colonization for group 3 patients only.

**Table 4:** Final logistic regression model for risk factors for fecal VRE colonization in kidney transplant patients.

Factors	Group of patients	Coefficient	Standard error	p		Likelihood ratio	95% confidence interval	
							Lower limit	Upper limit
CAPD	3	3.245	1.296	0.012		25.667	2.024	325.491
Hepatitis B	3	2.552	1.476	0.084		12.833	0.711	231.752
Vancomycin	3	2.041	0.844	0.016		7.700	1.471	40.293
Constant		-2.552	0.424	0.000				

CAPD = chronic ambulatory peritoneal dialysis.

## Discussion

VRE infection is a growing problem in specific groups of patients. However there is no data of VRE colonization prevalence in kidney transplant patients. Our study disclosed an unexpected high rate of VRE fecal colonization in such patients similar in the 3 groups of patients, in different pos-transplantation periods. This high rate cannot be compared to a healthy Brazilian population, since no data is currently available. On the other hand, very similar rates were observed in Brazilian risk groups [8,11].

In group 1 (less than 30 days after transplantation), fecal VRE carriage probably represented colonization within the dialysis setting. In this group, we excluded from analysis positive specimens that were collected more than 7 days apart because colonization could be associated with factors related to hospital admission, such as hospital transmission. In a simultaneous Brazilian study of 320 patients from an outpatient dialysis program, a prevalence rate of fecal VRE colonization of 14.4% was observed [8], very similar to overall rate we observed in transplant patients.

We found VRE prevalence rates of 15.1% in group 2 (1 to 6 months after transplantation) and 12.4% in group 3 (more than 6 months after transplantation), which are very similar to group 1 rates. This shows that the risk factors related to transplantation, such as the net state of immunosuppression, cumulative use of antimicrobial drugs and longer hospitalization (groups 2 and 3) were not related to increased VRE prevalence over time. The study design, however, did not allow us to evaluate whether VRE colonization was persisting or whether acquisition from outside the hospital was occurring. If the patients had been monitored using surveillance cultures during this six-month period, we would have been able to affirm with certainty whether colonization was persisting or not. On the other hand, it is possible that colonization persists over time, as has been described by others [9] and persistence may have been amplified as a consequence of the use of antimicrobial drugs or the net state of immunosuppression.

Our rates of fecal colonization were as high as those found in surveillance studies in Intensive Care Unit patients. Fridkin et al. [10], in a prospective study including 126 adult ICU patients in 60 American hospitals between January 1996 and July 1999, found an average prevalence rate of 10%. A similar rate has been disclosed in a single center Brazilian ICU study (14.5%) [11]. Such similar rates may be explained by the presence of similar risk factors for VRE acquisition, such as antimicrobial use, frequent and prolonged hospitalization and severity of underlying diseases.

Fifty percent of the VRE positive patients were colonized by *faecalis/faecium *species of *enterococci*. *E. gallinarum *is a species with intrinsic resistance to vancomycin and seems to be very frequently found in Brazilian studies, in contrast with other studies, and contributed to 28.9% of VRE positive samples in this study. Barbosa et al. [8] studying dialysis patients, observed that 57.1% of the VRE isolates were *E. gallinarum *and 10.7% were *E. casseliflavus*. Thus, our transplant patients seem to have more *faecalis/faecium *species of *enterococci *than patients on dialysis (50% vs. 28.6%). Camargo et al. [11], in a Brazilian ICU, found that 84% of VRE species recovered from fecal specimens of critical patients were *E. gallinarum*. These findings contrast with clinical disease due to *Enterococci*, since it is estimated that 80–90% of the human enterococcal infections are caused by *E. faecalis*, 10–15% by E. *faecium *and less than 5% by other species [12]. On the other hand, *E. casseliflavus and E. gallinarum *have recently been reported as causative agents of clinical disease [13,14]. Reid et al. [15] recently described 20 cases of bacteremia caused by *E. gallinarum or E. casseliflavus/flavescens*, which were observed in the Mayo Clinic between 1992 and 1998. It is not possible to affirm that this is a tendency and these unusual agents are becoming emerging pathogens in context of human disease.

Other studies have disclosed hemodialysis as an independent risk factor for VRE colonization, including an evaluation in our own dialysis facility [8,16] In fact, the first cases of VRE were documented in hemodialysis patients and an increasing prevalence of VRE colonization has been reported by Tokars et al (11–34%) in American hemodialysis centers between 1995 and 1999 [17]. Whether this reflects transmission within the dialysis facilities or intrinsic patient conditions has yet to be determined. Although CAPD was a risk factor for VRE colonization in group 3 this finding should be interpreted with great caution since only 3 patients from this group were on CAPD at the time of transplantation and the present study design differs from those that addressed the issue of dialysis and VRE colonization.

Concerning the use of post-transplantation vancomycin, it has been documented that there is a direct increase in VRE prevalence, not only in relation to dialysis but also in relation to transplantation [18,19]. Previous vancomycin use is a known risk factor for VRE colonization and ICU patients are particularly at risk. Recently, in an analysis of the association between VRE and previous use of antimicrobial drugs, Fridkin et al. [10] found a statistically significant association between VRE colonization and previous use of vancomycin among 126 adult ICUs in 60 American hospitals.

In our study, previous use of vancomycin was an independent risk factor for VRE colonization for Group 3 patients. In Group 2, out of the 73 patients studied, only two of them had received vancomycin and only one of these was VRE-positive (p = 0.28). In group 3, nine patients out of 105 received vancomycin. Association between vancomycin use and VRE colonization in this group may reflect the cumulative use of vancomycin, since the patients were followed for a longer period of time. Restriction of vancomycin use in kidney transplant patients has the clear advantage of preventing long term VRE fecal colonization.

More intense immunosuppression is clearly linked with higher rates of infection, especially with the use of high steroid doses for rejection treatment and anti-lymphocyte preparations [20,21]. Only one study showed a clear relationship between tacrolimus use and VRE colonization in a mixed group of infants with end stage renal disease [22]. We have not found an association between presumed more intense immunosuppression and VRE colonization, including variables such as CMV infection, pulses of methyl-prednisolone, OKT3 and MMF use. The net state of immunosuppression seems to play a role in VRE colonization, since higher rates are found in selected groups of immune impaired patients, mainly ICU patients. However, whether immune suppression is a risk factor itself or a surrogate marker for other risk factors remains to be determined. It is our impression that more intense immunosuppression among kidney transplant patients does not play a central role in VRE colonization.

Although there is a strong correlation between colonization and infection [23], during the study period we did not observe any case of VRE infection among the studied population. This must be explained by the fact that most patients were colonized in the outpatient (or were discharged shortly after colonization detection) setting where other risk factors commonly associated with VRE disease were not present.

This study has one important limitation. Two stool cultures were planned for all patients; however, this goal was achieved for 55 % of patients only although all efforts and infrastructure for patient access to collection sites were attempted were provided. This probably resulted in the disclosure of an underestimated colonization rate considering that 24 % of all positive patients had the first specimen negative and the second positive.

## Conclusion

VRE has become an important nosocomial pathogen because of its rapid spread, high mortality rates associated with infections, limited options for treatment and the possibility of transferring resistance genes to vancomycin to other more virulent and more prevalent pathogens such as *Staphylococcus aureus *[24]. Strategies to promptly identify colonized patients and apply contact precautions have led to lower endemic rates in some studies [24]. Prompt identification is based on targeted surveillance, considering risk factors for VRE colonization in selected patients, mainly hospitalized ICU patients. We observed an unexpectedly high rate of VRE colonization in kidney transplant patients, which is very similar to those observed in ICU's. Furthermore, a high rate of VRE colonization from outpatients was shown, raising the question of extending surveillance to recently admitted patients from the ambulatory setting. It is our opinion that due to cost concerns, this strategy could be reserved for patients with known risk factors (recent of frequent use of vancomycin) or to those with a higher probability of transmission, such as patients with diarrhea.

## Competing interests

The author(s) declare that they have no competing interests.

## Authors' contributions

MCSF carried out the acquisition of data and participated on design, analysis and interpretation of data.

APS participated in the revising of the manuscript.

DB participated in the design of the study.

SS carried out the microbiology studies.

HS participated in the revising of the manuscript

RS participated in the revising of the manuscript and the statistical analysis

LFAC conceived of the study, and participated in its design and coordination.

All authors read and approved the final manuscript.

## Pre-publication history

The pre-publication history for this paper can be accessed here:


